# Post-traumatic stress and depression following disaster: examining the mediating role of disaster resilience

**DOI:** 10.3389/fpubh.2024.1272909

**Published:** 2024-01-17

**Authors:** Jennifer M. First

**Affiliations:** College of Social Work, University of Tennessee, Knoxville, TN, United States

**Keywords:** disaster, mental health, resilience, depression, PTSD

## Abstract

The current study used structural equation modeling to examine the role of disaster resilience as a mediator between disaster exposure and post-traumatic stress and depressive symptoms among a sample of 625 U.S. adults who experienced a disaster event. Results found that disaster resilience mediated the relationship between disaster exposure as a predictor and depression and post-traumatic stress as dependent variables. These findings have important implications for understanding the mechanisms by which disaster resilience supports post-disaster mental health and can inform future disaster mental health interventions and practice models.

## Introduction

Environmental threats such as natural and human-caused disaster events (e.g., tornados, hurricanes, floods, oil spills) are increasing in prevalence and severity in the United States and worldwide. Between 2000 and 2019, approximately 510,837 individuals have died and 3.9 billion people have been affected by disasters (1). Disasters and other environmental threats pose profound risks to human well-being and cause widespread mortalities, morbidities, property loss, and reduced access to food, water, and housing (1). Furthermore, they can contribute to adverse psychological risks and behavioral health disorders, including substance use, depression, anxiety, and post-traumatic stress disorder (2, 3). After a disaster, it is common for people to experience a range of emotional and mental health difficulties, including stress, anxiety, fear, and grief. These effects can be short-term, such as increased stress and anxiety in the immediate aftermath of the disaster, or more long-term, such as the development of post-traumatic stress disorder (PTSD) and depression (3).

Various factors have been found to place individuals more at risk for developing depression and PTSD following disasters. Prior research [e.g., (4–6)] indicates the extent of psychological harm is associated with factors such as the severity of the disaster (e.g., EF-5 tornado), the degree of exposure (e.g., personal injuries, loss of home), and the magnitude of community destruction (e.g., the prevalence of homes, schools, and hospitals destroyed). For example, in a meta-analytic review, Brewin et al. (7) found an association between the severity of the disaster trauma (higher degree of disaster exposure) and the subsequent severity of depression symptoms. In addition, prior studies have indicated that a dose–response effect occurs, wherein PTSD and depression symptoms have been found to increase with greater disaster exposure levels (5, 8, 9).

While disaster exposure has been found to have a direct effect on post-disaster depression and PTSD, it could, however, also indirectly affect depression and PTSD through a third mediating variable, such as resilience. Although different definitions of resilience exist in the literature [for a review, see (10)], most of them generally share the idea that resilience is the ability of an individual to positively adapt in the face of stress, risk, and adversity (10–13). This definition indicates that resilience is a process and that protective factors (e.g., optimism, distress regulation, environmental resources) foster specific processes in the individual that assist in preventing adverse outcomes and promote positive adaption and growth following exposure to stressful or traumatic events (4, 14).

Within a disaster context, (15) described a risk and resilience framework, wherein resources or protective factors counterbalance the threats of disaster exposure. In terms of conceptualizing the process of resilience in a research model, resilience has the potential to operate as a mediator (16, 17) between risk factors (e.g., disaster exposure) and adverse outcomes [e.g., depression, PTSD; (18)]. Known as the “protective factor model,” resilience has been found to influence the effect of a risk factor by mediating the adverse impact of risk for predicting negative outcomes (19, 20). For example, in prior research, resilience has been found to mediate the relationship between interpersonal risk factors and hopelessness, and contribute to lower levels of hopelessness in a sample of individuals with clinical depression (19). Resilience has also been found to mediate COVID-19 pandemic-related stress and contribute to lower depression and anxiety (21), and higher academic success among college students (22).

Despite the role of resilience as a potential mediator between risk factors and mental health outcomes, few studies have examined the possible mediating relationship of resilience to mitigate against adverse mental health outcomes following exposure to disaster events (23). While there is a large amount of evidence that indicates disaster exposure and resource loss can have a detrimental impact on mental health after disasters (24, 25), however, less is known about the processes and mechanisms by which resilience mitigates risk factors and reduces the probability of a negative mental health outcome. Uncovering the potential mechanisms by which disaster resilience may be directly and indirectly related to mental health outcomes is important for disaster preparedness and response, as it can provide insights into protective factors that are particularly important in the event of a disaster. Therefore, to address this gap, the objective of the current study was to examine whether disaster resilience had a protective mediating effect on the relationship between disaster exposure and post-disaster depression and PTSD among 625 U.S. adults exposed to disaster (e.g., hurricane, tornado, wildfire, oil spill).

In the current study, disaster resilience was conceptualized as various internal and external factors that interact to influence an individual’s ability to adapt and recover following exposure to disaster (26). These results could provide a further understanding of the dynamic process of resilience by understanding its interactive mechanisms between exposure to disaster and post-disaster mental health. Structural equation modeling (SEM) was utilized to test this model, and a cross-sectional study was conducted among a sample of adults exposed to a disaster event (*N* = 625). Based on the evidence reviewed above, the following hypotheses guide this study:

*H1*. Disaster exposure will be positively associated with PTSD and depression.

*H2*. Disaster exposure will be positively associated with disaster resilience.

*H3*. Disaster resilience will (a) have an inverse or negative relationship with PTSD and depression and (b) will mediate the relationship between disaster exposure and PTSD and depression.

## Methods

### Data collection procedures

Data collection procedures were approved by the [Identity Removed for Review] Institutional Review Board (IRB). Participants qualified for this study if they were 18 or older and had experienced a disaster within the previous 3 years (2016–2019). To ensure the statistical analyses possessed sufficient statistical power with the SEM model, a power analysis was conducted to help determine the adequacy of the sample size required. The criterion was set that the estimated power needed to be 80% or higher, with a significance level (α) set at 0.05, for all the parameters of interest within the SEM (e.g., factor loadings, correlations, and regression paths), with a projected sample size of at least 500 participants was found as adequately powered (27).

Participants were recruited through purposive online sampling using Qualtrics’ panel aggregator sampling service. The Qualtrics panel aggregator provides researchers access to market research panels and uses digital technology (e.g., IP address checks, digital fingerprinting) to ensure participants’ data are as valid and reliable as possible (28). In addition, Qualtrics can monitor the data collection procedure and controls for issues such as participant inattentiveness or ineligibility, high incompletion rates, duplicate responses, or unreasonably quick completion times (29). Qualtrics was chosen as the online data collection platform following research indicating that samples recruited via online panel aggregators represent the U.S. population demography slightly better and are usually less expensive than convenience samples (30). Qualtrics invited participants to the study by clicking on a link to a screening questionnaire to assess eligibility if they lived in a U.S. state or territory that has experienced a natural or human-caused disaster in the prior 3 years (2016–2019). Accordingly, the states targeted for recruitment included California, Tennessee, North Carolina, South Carolina, Georgia, Alabama, Mississippi, Florida, and Texas. Using the online interface of Qualtrics, participants were provided with study instructions and self-reported questionnaire items. In addition, participants were compensated for their time with incentives through the Qualtrics incentive program (e.g., prize drawings and accumulated rewards).

### Measures

#### Disaster exposure

Disaster exposure (*M* = 9.72, *SD* = 1.72, *α* = 0.66) was measured by participants rating their perceptions of exposure to five main disaster-related stressors: did they lose personal belongs, was their home or property damaged, did they experience bodily injury, did their life or loved one’s life feel threatened, and did they experience feelings of helplessness, fear, or horror [see (31, 32)]. Participants rated their responses on a 4-point Likert scale with response options ranging from 1 = *not at all* to 4 = a *great deal*. All items were summed to create an observed variable.

#### Disaster resilience

Disaster resilience (*M* = 166.51, *SD* = 28.53, *α* = 0.96) was measured via the Disaster Adaptation and Resilience Scale [DARS; (26)], a 43-item scale consisting of five domains found to support disaster resilience, including: material resources, social resources, distress regulation, problem-solving, and optimism. Each item is rated on a 5- point Likert scale ranging from 0 (*not at all true*) to 4 (*true nearly all of the time*), with higher scores reflecting higher levels of resilience. Participants were prompted to think about the most recent disaster event and answer to report if they possess a specific protective factor (e.g., distress regulation, access to basic resources) on a 5-point Likert scale ranging from 0 = *not at all true* and 5 = *true nearly all the time* to create a latent variable.

#### Post-traumatic stress

Post-traumatic stress disorder (PTSD) symptoms (*M* = 34.76, *SD* = 23.22, *α* = 0.97) were measured via the Impact of Event Scale-Revised [IES-R; (33)]. The scale consists of three factors of symptoms related to posttraumatic stress: avoidance (eight items), hyperarousal (six items), and intrusion (eight items). Sample items include: “Any reminder brought back feelings about it,” “I felt irritable and angry,” and “My feelings about it were kind of numb.” In the current study, participants will be instructed to report how distressing or bothersome each symptom had been within the past 7 days with respect to the most recent disaster event. Responses for the IES-R are provided on a 5-point Likert-like scale which ranged from 1 = *not at all* to 5 = *extremely* to create a latent variable.

#### Depression

Depression (*M* = 3.93, *SD* = 1.97, *α* = 0.89) was measured via the Patient Health Questionnaire [PHQ-2; (34)]. The PHQ measures the degree to which an individual has experienced depressed mood over the past 2 weeks in order to screen participants for disaster-related depression. Responses were provided on a 4-point Likert-like scale which ranged from 0 = *not at all* to 3 = *nearly every day* to create a latent variable.

### Analyses

The demographic characteristics of respondents were analyzed using univariate methods including means, standard deviations, and frequencies. To examine the relationships between disaster exposure, disaster resilience, and mental health outcomes, structural equation modeling (SEM) was used. Using a two-step procedure recommended by Kline (35), first tested a measurement model (confirmatory factor analysis, CFA) to examine and confirm the factor structure of the latent variables and indicators (e.g., disaster resilience, PTSD, depression). Next, the structural model analyzed the direct effects of disaster exposure and mental health outcomes and whether the impact of disaster exposure on PTSD and depression, can be filtered or mediated by the individual’s level of disaster resilience.

For both the measurement and structural SEM models, a maximum likelihood estimation with robust standard errors was performed using R software and the *lavaan* package ((37, 38) R Development Core Team, 2011; Rosseel, 2012). Guidelines for goodness of fit indices were used to evaluate model fit based on the recommendations of Little (36) included the root mean square error of approximation (RMSEA) <0.08, standardized root mean square residual (SRMR) <0.08, and comparative fit index (CFI) > 0.90 and the Tucker-Lewis Index (TLI) > 0.90. Residuals were also inspected for outliers, which can indicate a model misfit that is not due to chance. In addition, modification indices were inspected for high values indicating the possible need to remove an item or change the path of an indicator (35). To test the mediation or indirect effects, the 95% confidence interval of 1,000 bootstrapped resamples of the product of coefficients were generated to ensure the confidence intervals do not include zero, and therefore the effect is considered statistically significant (37). In the case of missing data at random, a full information maximum likelihood estimation will be used, which assumes missing data points have an expectation equal to a model-derived value estimated from the remaining data points (38).

## Results

The final sample included 625 participants who experienced a disaster between 2016 and 2019. Missing data in the current study did not exceed 10% for any variable. Three hundred thirty participants were female (53%), and 293 were male (47%). The majority of participants identified as White (62.5%), followed by Black/African American/Afro Caribbean (*n* = 105, 16.8%), Hispanic/Latino (*n* = 55, 8.8%), Multi-racial (*n* = 29, 4.6%) Asian American (*n* = 26, 4.2%), Native Hawaiian/Other Pacific Islander (*n* = 9, 1.4%), and American Indian/Alaskan Native (*n* = 4, 0.6%). Nearly half of all participants had a bachelor’s degree or higher (*n* = 146, 49.7%). The average household size was 2.69. The most frequent disasters experienced by participants included hurricanes (*n* = 423, 68%), followed by tornados (*n* = 59, 9.5%), floods (*n* = 56, 9%), and wildfires (*n* = 54, 8.7%). See Table 1 for the complete descriptive statistics of the participants.

**Table 1:** Descriptive statistics.

Variable		*N*	%	*M*
Sex				
	Male	293	47	
	Female	330	53	
Race				
	American Indian/Alaskan Native	4	0.6	
	Black/African American/Afro-Caribbean	105	16.8	
	Native Hawaiian/Other Pacific Islander	9	1.4	
	Asian American	26	4.2	
	Hispanic/Latino	55	8.8	
	White	397	63.5	
	Multi-racial	29	4.6	
Age				32.49
Income				
	Less than $15,000	75	12	
	$15,000 to $29,999	130	15.6	
	$30,000 to $44,999	117	18.7	
	$45,000 to $59,999	91	14.6	
	$60,000 to $74,999	72	11.5	
	$75,000 to $104,999	61	9.8	
	$105,000 or more	79	12.6	
Education				
	Grade School	4	0.6	
	Some High School	17	2.7	
	High School Graduate	137	21.9	
	Some College	188	30.1	
	College Graduate	196	31.4	
	Advance Degree	80	12.8	
Disaster type				
	Hurricane	423	68	
	Tornado	59	9.5	
	Wildfire	54	8.7	
	Flood	56	9.0	
	Earthquake	12	1.9	
	Chemical Spill	4	0.60	
	Civil Unrest	7	1.0	
	Mass Shooting	4	0.60	

*N* = 625.

**Figure 1:**
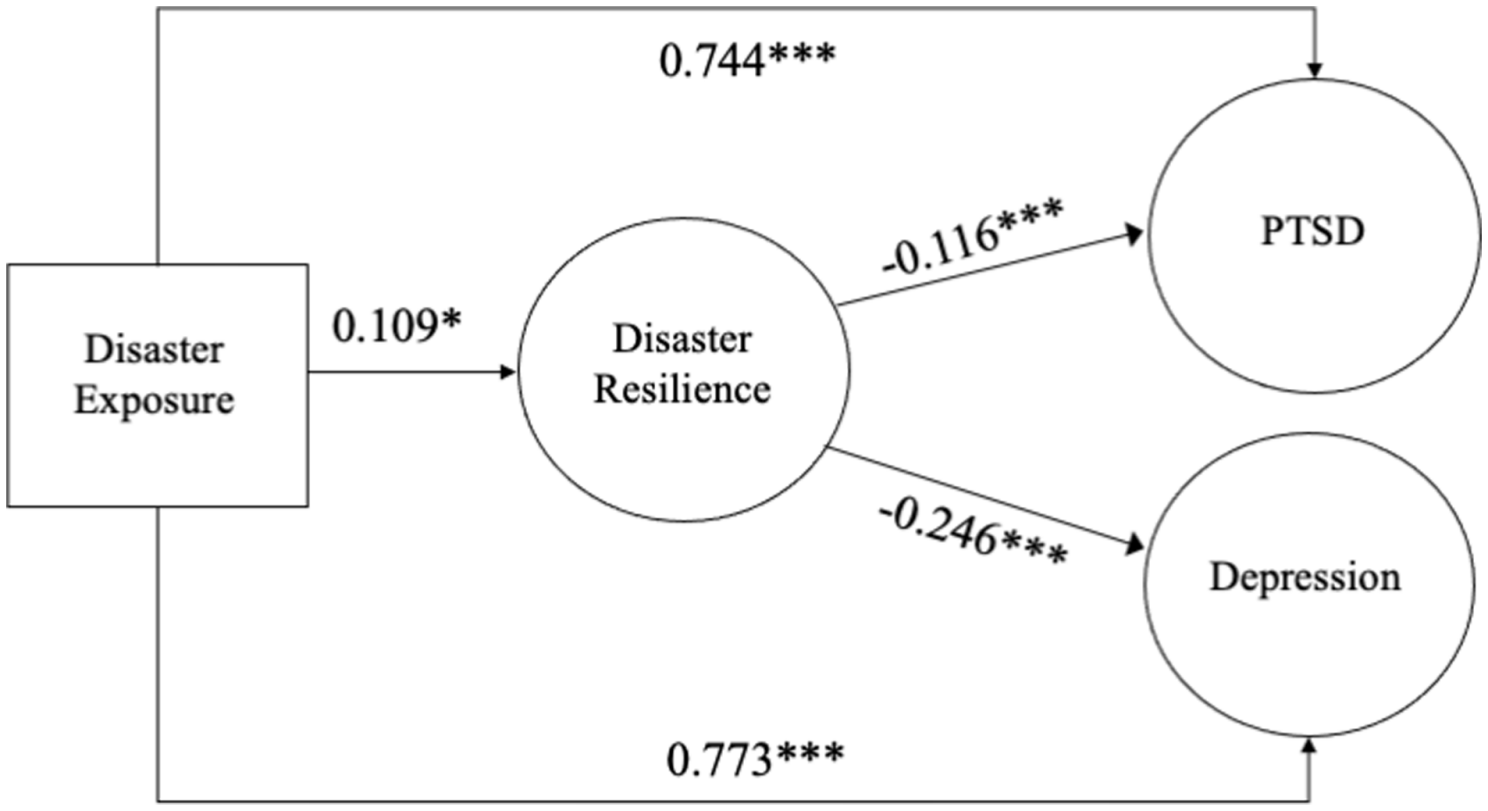
Diagram of structural model. *N*=625, Model Fit statistics: x^2^ (1117) = 2484.079, *p* < 0.01, CFI=0:91, TLI = 0.90, RMSEA=0.05, SRMR=0.06. PTSD= Posttraumatic stress symptoms. Solid lines with arrows indicate statistically significant. *p<.05, **p<.01, ***p<.001.

For the SEM analyses, a measurement model of the latent variables (e.g., disaster resilience, depression, PTSD) was first estimated and the initial measure model did not achieve an acceptable model fit as both the CFI and TLI were less than 0.90. To remedy this issue, parceling items, or combining indicators, can be a valuable method to improve model fit when latent variables have a high number of indicators and can provide information about the relationships among the latent variables (36). After parceling the 22 indicators for the PTSD latent variable into three equal-sized domain parcels, the measurement model achieved acceptable fit: χ2 (2108) = 4588.933, *p* < 0.01, CFI = 0.91, TLI = 0.90, RMSEA = 0.04, SRMR = 0.05. Next, the structural model was estimated and achieved acceptable fit, model fit statistics included χ2 (1117) = 2484.079, *p* < 0.01, CFI = 0.91, TLI = 0.90, RMSEA = 0.05, SRMR = 0.06, and allowed for the testing of hypotheses (Table 2 and Figure 1).

**Table 2:** Structural model: regression paths.

Regression paths	Unstandardized estimate	Standard error	Standard estimate
Disaster resilience (*R*^2^ = 0.12)			
Disaster exposure	0.202	0.010	0.109*
PTSD (*R*^2^ = 0.78)			
Disaster exposure	0.556	0.025	0.744***
Disaster resilience	−0.333	0.062	−0.116***
Depression (*R*^2^ = 0.62)			
Disaster exposure	0.255	0.016	0.773***
Disaster resilience	−0.395	0.060	−0.246***

Model Fit statistics: χ^2^ (1117) = 2484.079, *p* < 0.01, CFI = 0.91, TLI = 0.90, RMSEA = 0.05, SRMR = 0.06. PTSD = Posttraumatic stress symptoms. Solid lines with arrows indicate statistically significant.

*p < 0.05, **p < 0.01, ***p < 0.001.

In Figure 1, the SEM results revealed significant relationships among all the study variables. The first hypothesis (H1) predicted that disaster exposure would have a significant positive relationship with PTSD and depression symptoms. Results show that H1 was supported, and found that disaster exposure had a significant and positive relationship between PTSD (*β* = 0.744, *p* < 0.001) and depression (*β* = 0.773, *p* < 0.001). Individuals who had encountered more disaster-related losses and stressors (e.g., injuries, loss of a loved one, property damage) had a higher risk of disaster-related PTSD and depression. Next, the second hypothesis (H2) predicted that disaster exposure would have a significant and positive relationship with disaster resilience. Results found that H2 was supported and disaster exposure was significantly associated with having more disaster resilience (*β* = 0.109, *p* < 0.05). The increase in disaster exposure was found to predict an increase in the level of disaster resilience.

Finally, the third hypothesis (H3a) predicted that disaster resilience would be inversely associated with PTSD and depression symptoms. Results found that H3a was also supported as disaster resilience had a significant and negative association with PTSD (*β* = −0.116, *p* < 0.001) and depression (*β* = −0.246, *p* < 0.001). Furthermore, the third hypothesis (H3b), predicted that disaster resilience would mediate the relationship between disaster exposure and PTSD and depression. Results found that H3b was also confirmed as disaster resilience was to contribute to lower PTSD (*β* = −0.013, *p* < 0.05, [CI 95%: −0.028, −0.007]) and depression symptoms (*β* = −0.027, *p* < 0.01, [CI 95%: −0.046, −0.006]) based on the 95% confidence interval from 1,000 bootstrapped resamples.

## Discussion

Disaster events place stress on human life, livelihood, and health, and can have significant impacts on the mental health and well-being of individuals exposed. To test whether the impact of disaster exposure on PTSD and depression can be mediated by disaster resilience, this study examined direct and indirect relationships between disaster stress, disaster resilience, and mental health using structural equation modeling among 625 U.S. adults. Results from the current study point to several findings. First, SEM analysis found that individuals with more disaster exposure were associated with higher levels of PTSD and depressive symptoms. These findings are consistent with prior studies (2, 8, 41) indicating that individuals exposed to more disaster-related losses (i.e., property damage, injuries) were more likely to demonstrate symptoms of PTSD and depression, and illustrate that disaster exposure can have significant effects on the mental health of individuals.

Second, this study found that more exposure to disaster losses was associated with more resilience. This finding highlights that individuals experiencing greater amounts of disaster-related adversity required greater levels of resilience to help mitigate the negative effects of disaster exposure. Resilience or protective factors have been theorized to be able to help mitigate the effects of stressful and traumatic experiences after a collective trauma, and this study’s results confirm prior studies (15, 42) that have found a positive association between exposure to adversity contributing to greater resilience. However, researchers note that at certain doses, individuals may no longer be capable of adapting when exposure levels are cumulative and ongoing (8, 43, 44). For example, previous studies have found that cumulative exposure to multiple collective traumas may predispose people to negative mental health outcomes (43–45, 47). Future research should continue to examine the relationship between disaster exposure and resilience responses to time-limited stressor events and in the face of chronic, ongoing collective traumas (46).

In addition to acknowledging potential risks and adverse impacts from disasters, is an increased recognition and importance of understanding the mechanisms of disaster resilience (47). Results from this study found that disaster resilience demonstrated a significant mediating relationship between disaster exposure and PTSD and depression among participants. This finding provides further empirical support for conceptualizations of disaster resilience’s ameliorating role in contributing to better mental health outcomes following disaster exposure (49–51), and further theoretical understanding of the phenomena of resilience and how it operates in the specific context of disasters (Schneiderman et al., 2005). In other words, disaster resilience was found to play an important role in changing the strength or direction of the relationship between disaster exposure and post-disaster mental health, such that individuals with access to more disaster resilience (e.g., material, social, and psychological resources) contributed to fewer negative mental health effects. Understanding the underlying mechanisms that help to explain the relationships between risk factors and adverse outcomes provides important insights into potential inventions to target to improve disaster mental health response and preparedness. Findings from this study will be able to assist disaster researchers and practitioners in identifying protective factors (e.g., physical, social, and psychological resources) for intervention development that promote resilience and healthy psychological development in communities experiencing disaster.

Finally, these findings also have the potential to contribute to future research on identifying factors supporting the resilience of medical and healthcare professionals working in disaster and emergency response settings. Prior studies have found that working in disaster settings exposes healthcare workers to considerable stress, trauma, and emotional strain and can lead to conditions such as post-traumatic stress disorder (PTSD), depression, suicidality, and anxiety (52, 53), and this study illustrates the important mechanism or process of disaster resilience in reducing mental health symptoms. These findings could be used to inform future research on specific protective factors that could play a beneficial role in reducing negative mental health outcomes among high-risk medical workers in disaster settings (55). By systematically examining and refining these protective factors, future research can contribute to developing targeted interventions, training programs, and support systems tailored to the disaster resilience of the healthcare workforce.

## Limitations

In regard to study limitations, this project was limited by non-probability sampling, by self-report measures, cross-sectional design, and the sample’s disaster experiences (e.g., majority natural hazards, hurricanes). First, this study utilized non-probability sampling, and therefore, the results may not be generalizable to all individuals experiencing a disaster event. Future studies could improve on this limitation by utilizing a probability sampling design. Second, this study utilized self-report measures that may not be accurate as a full clinical evaluation of PTSD or depression symptomology. A third limitation is that this study is cross-sectional in design, and therefore, the collected data cannot make causal claims of temporal order (56). The current study’s cross-sectional limitation could be improved upon by future studies employing a longitudinal design that collects data at several points and could, for example, assess resilience at 1 month, 6 months, and 1 year to increase further knowledge about disaster resilience. Despite these limitations, this study found the presence of important associations that were consistent with theoretical predictions (e.g., disaster exposure and resilience had direct and indirect associations with PTS and depression symptoms).

## Conclusion

The current study used structural equation modeling (SEM) to identify the relationships between disaster exposure and disaster resilience on mental health outcomes in a sample of 625 U.S. adult participants. Results found that disaster exposure was significantly related to higher levels of PTS and depression symptoms. Disaster resilience was inversely related to PTSD and depression symptoms and played an important role in mediating the relationship between disaster exposure and mental health outcomes. Findings from this study can assist disaster researchers and practitioners in identifying protective factors to support disaster resilience interventions and practice models.

## Data availability statement

The raw data supporting the conclusions of this article will be made available by the authors, without undue reservation.

## Ethics statement

The studies involving humans were approved by University of Missouri IRB Board. The studies were conducted in accordance with the local legislation and institutional requirements. The participants provided their written informed consent to participate in this study.

## Author contributions

JF: Conceptualization, Formal analysis, Methodology, Writing – original draft, Writing – review & editing.
